# Analysis of prognostic factors affecting TA-TMA patients: a single-center retrospective study

**DOI:** 10.1007/s10238-025-01774-0

**Published:** 2025-07-04

**Authors:** Jile Liu, Haotian Meng, Tao Zhang, Xiu Liu, Mingfeng Zhao

**Affiliations:** 1 First Central Hospital of Tianjin Medical University, Tianjin Medical University, Tianjin, 300192 China; 2 Department of Hematology, Tianjin Thrombosis and Hemostasis Institute, Tianjin First Central Hospital, Tianjin, 300192 China; 3 Nankai University School of Medicine, Nankai University, Tianjin, 300071 China; 4 The Dean’s Office, Tianjin Xiqing District Zhongbei Town Community Health Service Center, Tianjin, 300380 China

**Keywords:** TA-TMA, Allogeneic hematopoietic stem cell transplantation, CAR-T, Creatinine, CNIs

## Abstract

**Objective:**

Transplantation-associated thrombotic microangiopathy (TA-TMA) is a severe thrombotic complication that occurs after hematopoietic stem cell transplantation. Currently, there are no clear conclusions on the factors influencing the prognosis of TA-TMA patients and the best treatment plan for TA-TMA.

**Methods:**

We retrospectively collected information on 26 patients with TA-TMA from a single center. The independent factors affecting prognosis were analyzed through univariate and multivariate Cox regression. Subgroup analysis was also conducted to investigate the impact of different treatment methods on overall survival of patients.

**Results:**

We found that high creatinine levels during the diagnosis of TA-TMA and previous CAR-T cell therapy were independent risk factors affecting the prognosis of TA-TMA patients. Additionally, discontinuing calcineurin inhibitors (CNI) is helpful in prolonging the survival period of TA-TMA patients.

**Conclusions:**

Our research has revealed the high-risk factors affecting the survival of TA-TMA patients and the efficacy of different treatment methods, providing guidance for subsequent clinical practice.

**Supplementary Information:**

The online version contains supplementary material available at 10.1007/s10238-025-01774-0.

Dear Editor,

Transplantation-associated thrombotic microangiopathy (TA-TMA) is a serious complication after allogeneic hematopoietic stem cell therapy (allo-HSCT), with a high non recurrent mortality (NRM) rate. The mortality rate of TA-TMA patients who are not treated in a timely manner is 50–90%, and the mortality rate of high-risk patients can reach 80% [1]. At present, the adverse prognostic factors and treatment of TA-TMA are still unclear and requires extensive research. Therefore, we retrospectively collected information on patients who were considered to have TA-TMA after receiving allogeneic hematopoietic stem cell transplantation at the Hematology Department of Tianjin First Central Hospital from March 2021 to July 2024. The follow-up endpoint is patient death or July 31, 2024. Supplementary Table 1 lists the five diagnostic criteria for TA-TMA, among which the diagnostic criteria proposed by Jodele et al. are most recognized for their practicality and comprehensiveness [2]. Therefore, the diagnostic criteria for TA-TMA in this study were based on the criteria proposed by Jodele et al [2].

From March 2021 to July 2024, a total of 274 patients in the Hematology Department of Tianjin First Central Hospital received allogeneic hematopoietic stem cell transplantation. Among them, 26 patients were diagnosed with TA-TMA after receiving allo-HSCT. The incidence rate of TA-TMA was 9.49%. The median number of days from allo-HSCT to TA-TMA occurrence was 53.5 days (14–256 days). Through univariate Cox regression analysis, we found that factors such as gender, number of previous treatment lines, concurrent occurrence of severe graft-versus-host disease, and liver function indicators had no statistically significant impact on the prognosis of TA-TMA patients. In univariate analysis, previous chimeric antigen receptor T cell (CAR-T) therapy, infection status at TA-TMA diagnosis, ferritin levels at TA-TMA diagnosis, and creatinine levels at TA-TMA diagnosis were associated with patient survival (Table 1). In multivariate analysis, there was still a significant difference in creatinine levels (HR 1.011, 95% CI 1.003–1.020, p = 0.008) and previous received CAR-T therapy (HR 4.265, 95% CI 1.304–13.955, p = 0.016). Therefore, we believe that high creatinine levels during TA-TMA diagnosis and previous CAR-T therapy are independent risk factors affecting the prognosis of TA-TMA patients.

**Table 1:** The impact of clinical characteristics on the survival of TA-TMA patients

Variables	Univariate Cox regression analysis		
	HR	95% CI	P value
Age	1.012	0.983–1.042	0.419
Sex	1.281	0.463–3.547	0.633
Lines of therapy	1.108	0.930–1.321	0.252
Previously received CAR-T treatment	3.049	1.047–8.880	0.041
Blood types between donor and recipient	1.912	0.587–6.228	0.282
Gender between donor and recipient	1.718	0.577–5.121	0.331
Infection status upon diagnosis of TA-TMA	2.543	0.884–7.315	0.083
Ferritin levels at diagnosis of TA-TMA	1.004	1.000–1.008	0.036
AST levels at diagnosis of TA-TMA	1.000	0.999–1.001	0.640
ALT levels at diagnosis of TA-TMA	1.000	0.998–1.001	0.910
Creatinine level at diagnosis of TA-TMA	1.007	1.000–1.015	0.051
GVHD at diagnosis of TA-TMA	1.614	0.452–5.757	0.461

Variables	Multivariate Cox regression analysis		
	HR	95% CI	*P* value
Previously received CAR-T treatment	4.265	1.304–13.955	0.016
Infection status upon diagnosis of TA-TMA	1.900	0.571–6.321	0.295
Ferritin levels at diagnosis of TA-TMA	1.003	0.999–1.008	0.130
Creatinine level at diagnosis of TA-TMA	1.011	1.003–1.020	0.008

*TA-TMA* Transplantation-associated thrombotic microangiopathy, *CAR-T* Chimeric antigen receptor T therapy, *GVHD* Graft-versus-host disease

We have chosen effective treatment methods reported in different clinical trials for TA-TMA patients [3]. Supplementary Fig. 1 shows the specific treatment measures and outcomes of the 26 patients included starting from allogeneic hematopoietic stem cell transplantation. Based on subgroup analysis, we evaluated the impact of specific treatment methods on overall survival of TA-TMA patients. Therapeutic plasma exchange (TPE) can help improve patients' symptoms. Among the 17 TA-TMA patients who received TPE, 11 patients' clinical indicators returned to normal. However, TPE cannot change the prognosis (Supplementary Fig. 2A). Withdrawing calcineurin inhibitors (CINs) can help prolong the survival of TA-TMA patients (Supplementary Fig. 2B). However, the use of eculizumab may not be beneficial for the prognosis of patients (Supplementary Fig. 2C). There was no statistically significant difference in patient survival between combination therapy and monotherapy (Supplementary Fig. 2D).

TA-TMA is an extremely serious complication of allo-HSCT, presenting patients with a dangerous and complex condition. Exploring the risk factors and optimal treatment plans that affect the prognosis of TA-TMA patients has become a current focus of research. This study firstly discovered that previous CAR-T therapy is one of the independent risk factors for poor prognosis in TA-TMA patients. CAR-T therapy is an effective measure for treating recurrent/refractory hematologic malignancies, but it also has serious side effects such as cytokine release syndrome (CRS) and immune effector cell-associated neurotoxicity syndrome (ICANS) [4]. During the process of killing tumors, CAR-T cells often overstimulate the immune system, leading to the release of a large amount of cytokines and causing damage to normal tissues and blood vessels [5, 6]. Among the patients we evaluated who had previously received CAR-T therapy, all patients experienced varying degrees of CRS. TA-TMA patients who have received CAR-T therapy have a poor prognosis, which may be related to the damage to vascular endothelial cells caused by CAR-T therapy. In recent clinical reports, it has also been mentioned that there is a correlation between IL-6 and TA-TMA occurrence [7]. Therefore, patients who have received CAR-T therapy before allo-HSCT should pay close attention, regularly test TA-TMA related diagnostic indicators, and detect and treat early. In addition, the inflammatory cytokine storm caused by CAR-T cells infusion should be corrected promptly to reduce the damage of inflammatory cytokines to vascular endothelium.

## Supplementary Information

Below is the link to the electronic supplementary material.

Supplementary file 1 (DOC 16631 KB)

## Data Availability

No datasets were generated or analyzed during the current study.
