# Supplementary material for: Analysis of prognostic factors affecting TA-TMA patients: a single-center retrospective study
Source: Clin Exp Med. 2025 Jul 4;25(1):232. doi: 10.1007/s10238-025-01774-0 (PMC12226656; doi:10.1007/s10238-025-01774-0)
Supplement: Supplementary file 1 — (DOC 16631 KB) [file 10238_2025_1774_MOESM1_ESM.doc]

Supplementary Table 1 Five different diagnostic criteria for TA-TMA.

| Jodele et al.^1^ | Cho et al. | BMT CTN | IWG | City of Hope |
| --- | --- | --- | --- | --- |
| LDH＞ULN | LDH＞ULN | LDH＞ULN | LDH＞ULN | LDH＞2ULN |
| Proteinuria (Random urinalysis protein ≥30 mg/dL or rUPCR ≥2 mg/mg） | Coombs’ test negative | Coombs’ test negative | Hemoglobin decrease or requiring transfusion RBC | Evidence of microangiopathy (schistocytes appear in  peripheral blood) |
| Hypertension | Without  coagulopathy | Renal and/or neurologic failure without cause | Serum haptoglobin decrease | sCr >1.5 ULN |
| Thrombocytopenia (PLT＜50 × 10^9^ /L or ≥50% decrease in count) | Thrombocytopenia (PLT＜50 × 10^9^ /L or ≥50% decrease in count) | Evidence of microangiopathy (peripheral smear ≥2 schistocytes/HPF) | Thrombocytopenia (PLT＜50 × 10^9^ /L or ≥50% decrease in count) | Thrombocytopenia (PLT＜50 × 10^9^ /L or ≥50% decrease in count) |
| Anemia (hemoglobin lower than limit or requiring transfusion RBC) | Hemoglobin decrease |  | Evidence of microangiopathy (≥4% schistocytes in peripheral blood) |  |
| Evidence of microangiopathy (schistocytes appear in  peripheral blood or having  histologic evidence) | Evidence of microangiopathy (peripheral smear ≥2 schistocytes/HPF) |  | Without  coagulopathy |  |
| Complement activation (sC5b-9 ＞ULN) | Serum haptoglobin decrease |  |  |  |
| ^1^Patients can be diagnosed with the evidence of microthrombus in tissue biopsy or with 5 or more of the following 7 conditions  LDH: lactate dehydrogenase; ULN: upper limit of normal; rUPCR: random urine protein/creatinine ratio; PLT: platelet; RBC: red blood cell; HPF: high power field; sCr: serum creatinine | | | | |

Supplementary Table 2 Basic information of the patients.

| No | Gender | | Age | Diagnosis | Previous treatment lines before allo-HSCT | previous CAR-T treatment history | Graft type | Blood types between donor and recipient | Gender between donor and recipient | Severe GVHD at diagnosis of TA-TMA | TA-TMA  diagnosis  days post allo-HSCT | Schistocytes | LDH | sC5b-9 (ng/ml) | Hb (g/L) | PLT (×10^9^/L) | Proteinuria (+) |
| --- | --- | --- | --- | --- | --- | --- | --- | --- | --- | --- | --- | --- | --- | --- | --- | --- | --- |
| 1 | Male | | 30 | MDS | 1 | No | haplo-HSCT | Same | No | Yes | 256 | - | 583.7 | 638 | 68 | 16 | 0 |
| 2 | Female | | 19 | ALL | 2 | No | haplo-HSCT | Different | Yes | Yes | 38 | + | 369.6 | 649 | 67 | 27 | 0 |
| 3 | Female | | 61 | MDS | 2 | No | haplo-HSCT | Different | Yes | Yes | 49 | - | 690.2 | 584 | 84 | 10 | 1+ |
| 4 | Male | | 31 | AML | 7 | Yes | haplo-HSCT | Same | Yes | Yes | 32 | - | 577 | 516 | 75 | 41 | 1+ |
| 5 | Male | | 37 | ALL | 5 | Yes | haplo-HSCT | Same | No | Yes | 55 | - | 780.2 | 540 | 59 | 15 | 2+ |
| 6 | Male | | 65 | MDS | 4 | No | HLA-matched | Same | Yes | Yes | 78 | - | 249 | 316 | 73 | 24 | 1+ |
| 7 | Male | | 31 | MDS | 1 | No | haplo-HSCT | Same | Yes | Yes | 25 | - | 300 | 249 | 79 | 14 | 2+ |
| 8 | Female | | 31 | AML | 3 | No | haplo-HSCT | Different | No | Yes | 31 | - | 269.9 | 230.5 | 72 | 64 | 2+ |
| 9 | Female | | 19 | ALL | 5 | Yes | haplo-HSCT | Same | No | Yes | 88 | - | 278.5 | 259.5 | 62 | 27 | 3+ |
| 10 | Female | | 62 | ALL | 5 | Yes | haplo-HSCT | Same | Yes | Yes | 52 | + | 569.1 | 391.8 | 60 | 1 | 2+ |
| 11 | Female | | 17 | AML | 8 | No | haplo-HSCT | Different | Yes | No | 120 | + | 680 | 409 | 58 | 5 | 0 |
| 12 | Female | | 33 | ALL | 4 | No | haplo-HSCT | Same | Yes | Yes | 200 | + | 620.7 | 408 | 74 | 19 | 1+ |
| 13 | Male | | 34 | MDS | 2 | No | HLA-matched | Same | Yes | Yes | 51 | - | 663 | 911 | 57 | 6 | 1+ |
| 14 | Female | | 44 | AML | 1 | No | haplo-HSCT | Different | Yes | Yes | 70 | - | 315.5 | 301 | 99 | 63 | 0 |
| 15 | Female | | 56 | AML | 4 | No | haplo-HSCT | Different | No | No | 28 | - | 884.2 | 194.1 | 76 | 16 | 2+ |
| 16 | Female | | 6 | AA | 1 | No | haplo-HSCT | Different | No | Yes | 18 | + | 1169 | 546 | 106 | 60 | 2+ |
| 17 | Male | | 55 | AML | 6 | No | haplo-HSCT | Different | Yes | Yes | 78 | - | 689.9 | 599 | 103 | 28 | 2+ |
| 18 | Female | | 26 | AML | 5 | Yes | haplo-HSCT | Different | No | Yes | 137 | - | 1219.8 | 477 | 77 | 2 | 2+ |
| 19 | Female | | 39 | MDS | 1 | No | haplo-HSCT | Same | Yes | No | 69 | - | 497.1 | 243 | 80 | 15 | 2+ |
| 20 | Male | | 43 | AML | 4 | No | haplo-HSCT | Same | Yes | Yes | 69 | - | 762.2 | 566 | 76 | 19 | 1+ |
| 21 | Female | | 58 | MPAL | 6 | Yes | haplo-HSCT | Different | Yes | No | 14 | + | 638.3 | 2582 | 62 | 2 | 1+ |
| 22 | Male | | 17 | ALL | 5 | Yes | haplo-HSCT | Different | Yes | Yes | 20 | - | 458.6 | 591 | 46 | 13 | 1+ |
| 23 | Female | | 37 | ALL | 1 | No | haplo-HSCT | Different | No | No | 30 | - | 430.8 | 444.8 | 79 | 34 | 0 |
| 24 | Male | | 27 | AML | 1 | No | haplo-HSCT | Same | Yes | No | 64 | - | 763.3 | 232 | 123 | 30 | 0 |
| 25 | Female | | 55 | AML | 3 | No | haplo-HSCT | Same | Yes | Yes | 46 | - | 615.9 | 591 | 78 | 5 | 0 |
| 26 | Male | | 16 | AA | 1 | No | haplo-HSCT | Different | Yes | Yes | 87 | - | 689.5 | 241.2 | 98 | 51 | 2+ |
|  | | GVHD, graft versus host disease; TA-TMA, transplantation-associated thrombotic microangiopathy; allo-HSCT, allogeneic hematopoietic stem cell transplantation; CAR-T, chimeric antigen receptor T cell; LDH, lactate dehydrogenase; Hb, hemoglobin; PLT, platelet; haplo-HSCT, haploidentical hematopoietic stem cell transplantation | | | | | | | | | | | | | | | |


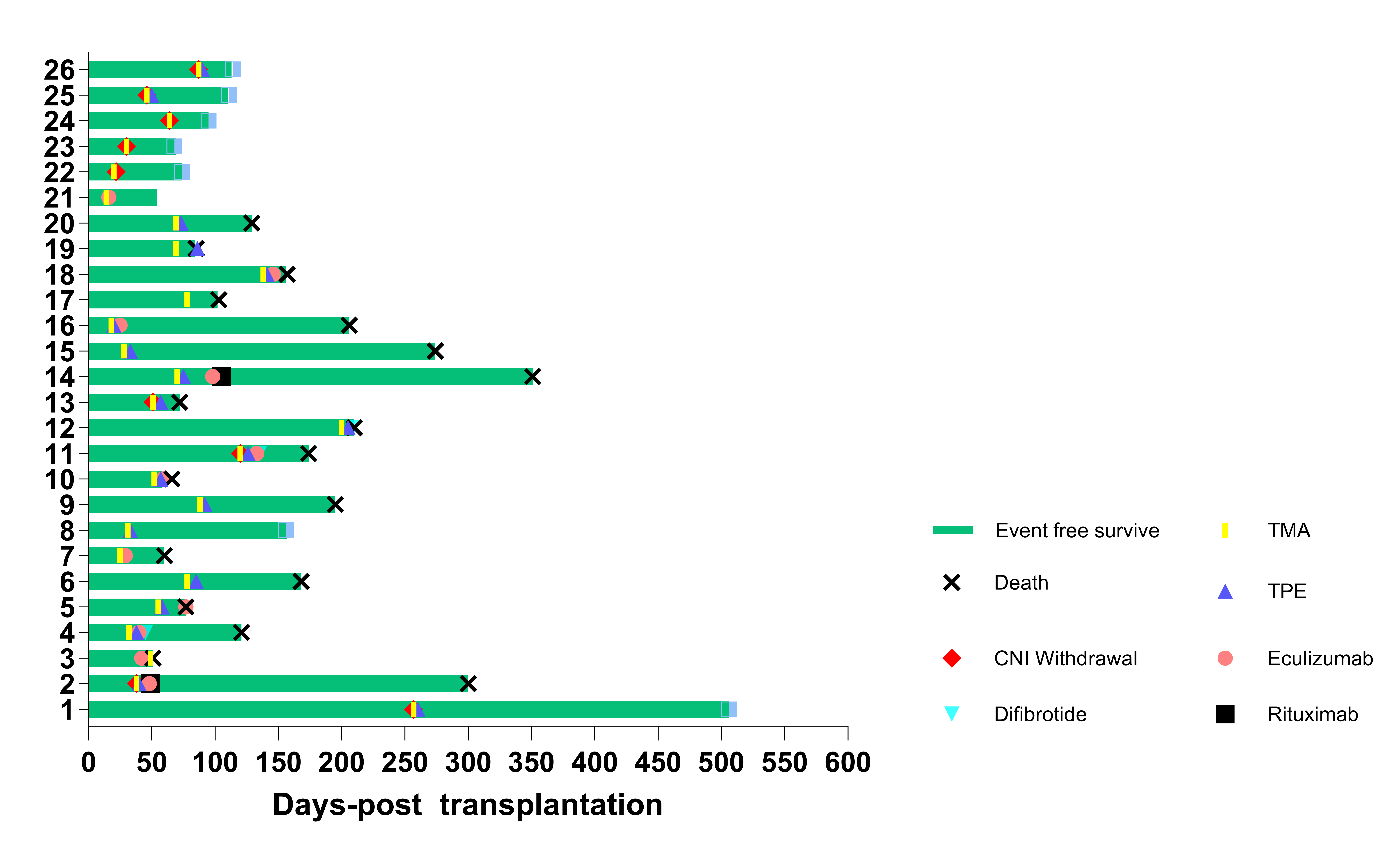


Supplementary Figure 1 Clinical outcomes of 26 TA-TMA patients receiving different treatments after allo-HSCT.


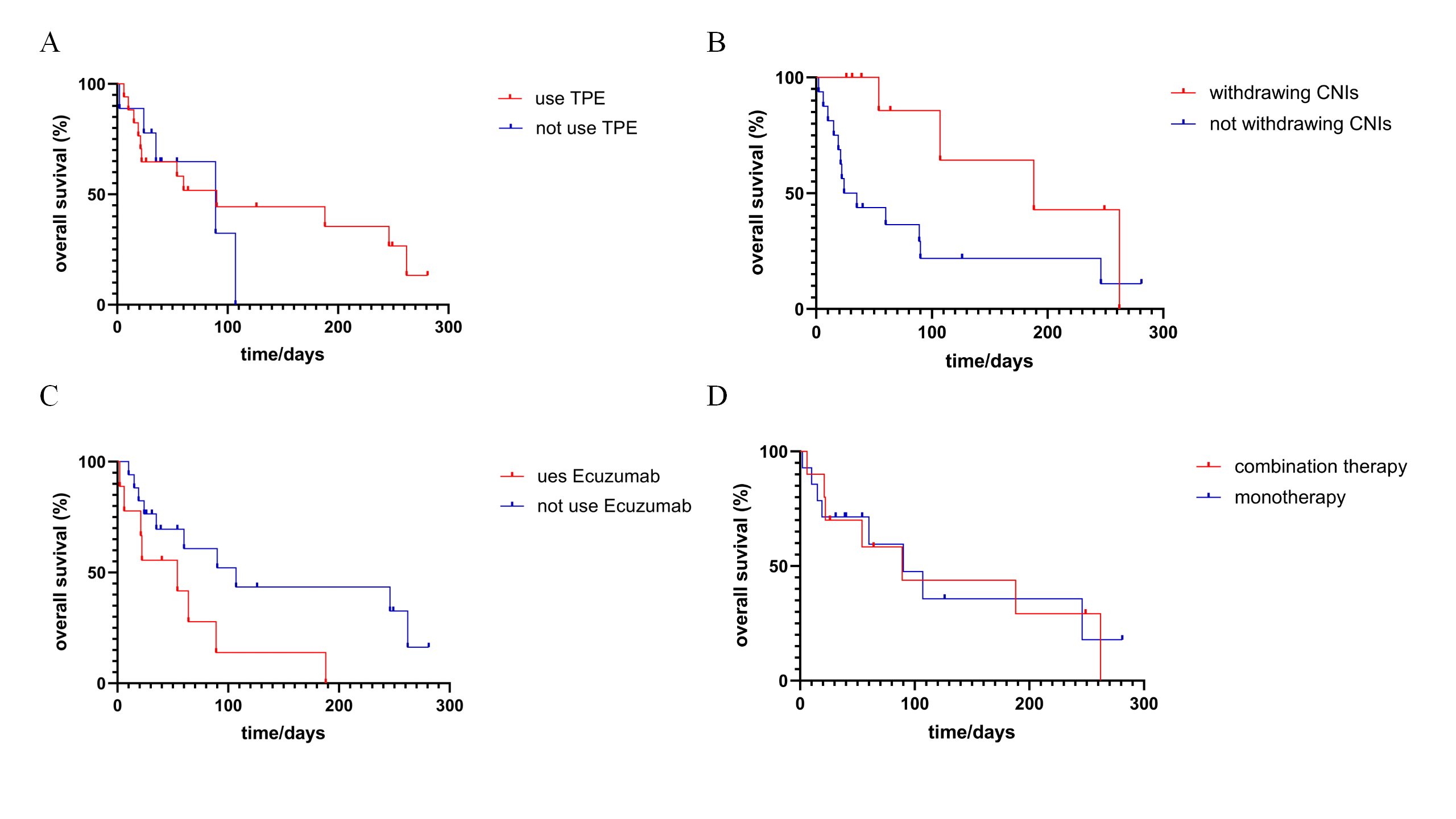


Supplementary Figure 2 Subgroup analysis of overall survival in TA-TMA patients.

1. The impact of TPE use on overall survival of TA-TMA patients.P=0.6292；B. The impact of withdrawing CNIs on overall survival of TA-TMA patients.P=0.0401;C. The impact of using Eculizumab on overall survival of TA-TMA patients.P=0.0389;D. The impact of monotherapy and combination therapy on overall survival of TA-TMA patients.P=0.9258.
